# Inhibiting HSD17B8 suppresses the cell proliferation caused by PTEN failure

**DOI:** 10.1038/s41598-024-63052-5

**Published:** 2024-05-29

**Authors:** Wei Zhao, Ruiting Huang, Dongyang Ran, Yutong Zhang, Zhi Qu, Shanqing Zheng

**Affiliations:** 1 School of Nursing and Health, Henan University, Kaifeng, Henan Province China; 2 School of Basic Medical Sciences, Henan University, Kaifeng, Henan Province China; 3 Laboratory of Cell Signal Transduction, Henan Provincial Engineering Centre for Tumor Molecular Medicine, Medical School of Henan University, Kaifeng, Henan Province China

**Keywords:** *C. elegans*, MCF-7, PTEN, *Daf-18*, *F12E12.11*, Cell proliferation, Cancer, Cell biology, Health care

## Abstract

Loss of the tumor suppressor *PTEN* homolog *daf-18* in *Caenorhabditis elegans (C. elegans*) triggers diapause cell division during L1 arrest. While prior studies have delved into established pathways, our investigation takes an innovative route. Through forward genetic screening in *C. elegans*, we pinpoint a new player, *F12E12.11*, regulated by *daf-18*, impacting cell proliferation independently of PTEN's typical phosphatase activity. F12E12.11 is an ortholog of human estradiol 17-beta-dehydrogenase 8 (HSD17B8), which converts estradiol to estrone through its NAD-dependent 17-beta-hydroxysteroid dehydrogenase activity. We found that PTEN engages in a physical interplay with HSD17B8, introducing a distinctive suppression mechanism. The reduction in estrone levels and accumulation of estradiol may arrest tumor cells in the G2/M phase of the cell cycle through MAPK/ERK. Our study illuminates an unconventional protein interplay, providing insights into how PTEN modulates tumor suppression by restraining cell division through intricate molecular interactions.

## Introduction

L1 (first larval stage, L1) diapause in *Caenorhabditis elegans* (*C. elegans*) is widely used as a model to study the roles of nutrient sensing and signal transduction in development^1,2^. Worms hatched in the absence of food maintain a quiescent state after embryonic development. However, loss of *daf-18* (human PTEN homology gene in *C. elegans*) causes the worms to escape from the diapause state, leading to cell proliferation even in the absence of food^3^. Quiescent M cell, germ cells, and Q cells start to divide in *daf-18*(−) L1 arrested worms^3–6^. Cell division caused by *daf-18* loss in L1-arrested worms is governed by branched signaling cascades^2^. Previously, we reported that Q cell division in *daf-18(−)* L1 arrested worms is regulated by a downstream RAF-MAPK (rapidly accelerated fibrosarcoma, RAF; mitogen-activated protein kinase, MAPK) signaling pathway^6^; however, the intermediary signals regulated by *daf-18* loss to activate MAPK still need to be identified. In this study, forward genetic screening was used to identify mutants with the ability to reverse the phenotype caused by *daf-18* loss^7^. A frameshift deletion in *F12E12.11* (also known as *nta-1*) was identified to suppress Q cell proliferation in *daf-18*(−) mutants. *F12E12.11* is predicted to exhibit oxidoreductase activity and is an ortholog of human estradiol 17-beta-dehydrogenase 14 (HSD17B14, Wormbase). However, when the protein sequence of F12E12.11 was blasted using BLASTP (National Center for Biotechnology Information, NCBI), the best match found in *H. sapiens* (Human) was estradiol 17-beta-dehydrogenase 8 (HSD17B8). Both HSD17B8 and HSD17B14 convert estradiol (E2) to estrone (E1) through NAD-dependent (Nicotinamide adenine dinucleotide, NAD) 17-beta-hydroxysteroid dehydrogenase activity *in vitro*^8,9^. Estrogens are the most important factors associated with the risk of developing breast cancer, and more than half of breast cancers are estrogen receptor positive (ER +)^10,11^. A high intratumor E1:E2 ratio resulting from the activity of HSD17B14 was reported to increase the number of tumor-initiating stem cells and ER + breast cancer growth *in vivo*^12^. However, HSD17B8, not HSD17B14, was found to affect the activity of MAPK in our study. HSD17B8 might be an important transduction factor in the DAF-18-MAPK signaling cascade. DAF-18 is an ortholog of human PTEN (phosphatase and tensin homolog)^13–15^. How HSD17B8, as a dehydrogenase, is regulated by the phosphatase DAF-18/PTEN to regulate cell proliferation in vivo has not been reported. In our study, we found that deletion of *F12E12.11* suppressed cell proliferation caused by *daf-18* loss in L1-arrested worms. Moreover, knocking down the HSD17B8 ortholog suppressed ER + breast cancer cell proliferation. We found that the PTEN protein can interact with HDS17B8 in cells to prevent HSD17B8 from converting E2–E1. Loss of PTEN results in the release and activation of HSD17B8 to consume E2. A reduction in E2 levels and accumulation of E1 may lead to ERK/MAPK (extracellular signal-regulated kinase 1/2, ERK) activation to inhibit ER + breast cancer cell growth. Our study provides a new mechanism by which PTEN suppresses cell proliferation, with PTEN inhibiting the enzyme function of HDS17B8 by interacting with it rather than phosphorylating it.

## Results and discussion

### *F12E12.11* loss can block Q cell proliferation in L1-arrested daf-18(−) worms

**Figure 1:**
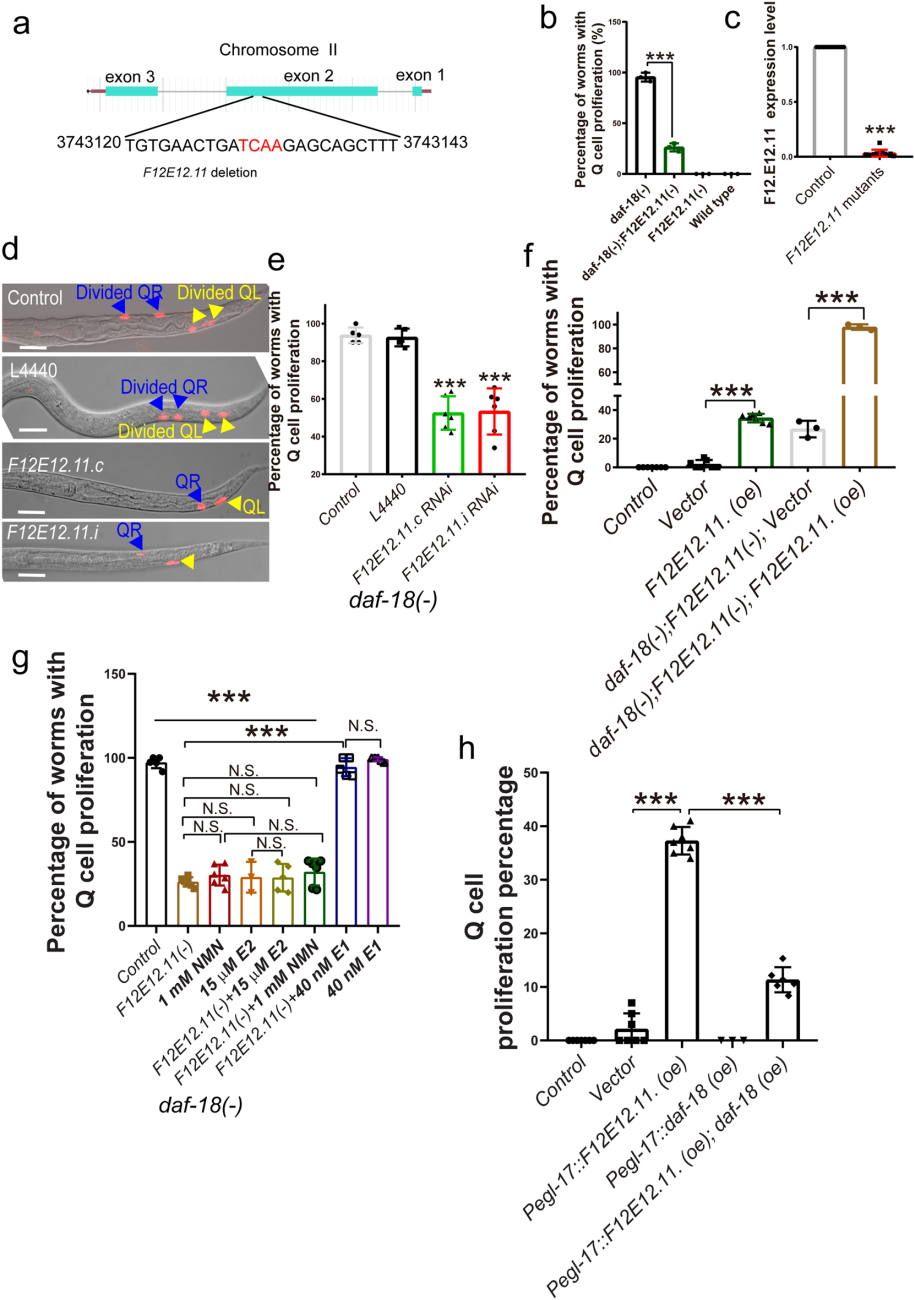
*F12E12.11* plays a role in Q cell proliferation in L1-arrested worms. (**a**) Whole-genome sequencing revealed that the “TCAA” sequence in the second exon of *F12E12.11* was deleted in *F12E12.11* mutants generated by EMS mutagenesis. The deletion was a frame-shift deletion that resulted in an early stop codon. (**b**) Q cell proliferation is suppressed by *F12E12.11* mutation. (**c**) The gene expression level of *F12E12.11* in *F12E12.11* mutants generated by EMS mutagenesis. The test was repeated at least 3 times, error bar: mean ± SEM. (**d**–**e**) Q cell proliferation was tested by knocking down *F12E12.11* in *daf-18*(−) mutants. (**f**) Q cell proliferation caused by overexpression of *F12E12.11* in wild-type and *daf-18*(−); *F12E12.11*(−) worms. (**g**) Effects of *F12E12.11,* E1, E2, and NMN on Q cell proliferation. (**h**) Overexpression of *daf-18* in neurons suppressed Q cell proliferation caused by *F12.E12.11*. N.S.: no significant difference. ***: P < 0.001, t-test. (**b**, **d**–**h**) The test was repeated at least 3 times, with a sample size larger than 60 in each repeat, error bar: mean ± SEM.

Loss of *daf-18* results in Q cell proliferation in all L1-arrested worms^6^. To identify the target genes of *daf-18*, EMS (ethyl methanesulfonate, EMS)-mutagenized *daf-18*(−) animals were screened for mutations that can suppress Q cell division using a classic forward genetic screening method^16,17^. After testing more than a thousand F3 generation EMS-mutagenized daf-18 worms, we found that L1-arrested *daf-18*(−) worms with a frameshift deletion in *F12E12.11* (Fig. 1a) exhibited lower Q cell proliferation (Fig. 1b). This frameshift deletion in *F12E12.11* resulted in a premature termination codon, which may have caused a reduction in mRNA levels. To test whether *F12E12.11* mRNA levels were decreased, real-time PCR was performed to measure the expression of *F12E12.11*. We found that the gene was barely expressed in these mutants (Fig. 1c). According to the ORFeome project (REF), there are two forms of *F12E12.11* with open reading frames (ORFs): *F12E12.11.i* and *F12E12.11.c.* We used two distinct, isoform-specific RNAi targeting constructs to knock down these two forms of *F12E12.11* in *daf-18*(−) mutants and found that the percentage of proliferating Q cells was significantly reduced in these RNAi-treated L1-arrested worms (Fig. 1d–e). Then, the wildtype *F12E12.11* sequence driven by its own promoter was used to rescue the suppression in *daf-18*(−); *F12E12.11*(−) mutants. We found that putting back *F12E12.11* abolished the suppression of Q cell divisions in *daf-18*(−) L1 arrested worms (Fig. 1f). To further confirm the function of *F12E12.11* in Q cell proliferation, we tested whether *F12E12.11* can induce Q cell proliferation in wild-type L1-arrested worms by overexpressing *F12E12.11* in N2 worms. Our results showed that the *F12E12.11*-overexpressing L1-arrested worms showed a higher percentage of proliferating Q cells (Fig. 1f). *F12E12.11* is predicted to convert E2 to estrone E1 through NAD-dependent oxidoreductase activity. We treated *daf-18*(−) and *daf-18*(−); *F12E12.11*(−) worms with E2, E1, and NAD + (Nicotinamide adenine dinucleotide, NAD +) precursor nicotinamide mononucleotide (NMN). Our results showed that *F12E12.11*(−), E2, and NMN all can significantly suppress cell proliferation, and E2 and NMN treatments have no additional suppression effects on *F12E12.11*(−), and E1 treatment can significantly induce cell proliferation even in *F12.E12.11*(−) mutants (Fig. 1g). Furthermore, the Q cell proliferation caused by overexpression *F12.E12.11* can be suppressed by *daf-18* (Fig. 1h). These results suggest that *F12E12.11* increases Q cell division in L1-arrested worms and that *F12E12.11* deletion can suppress Q cell proliferation caused by the loss of *daf-18* during L1 arrest.

### *F12E12.11* works through mpk-1 to control Q cell division

**Figure 2:**
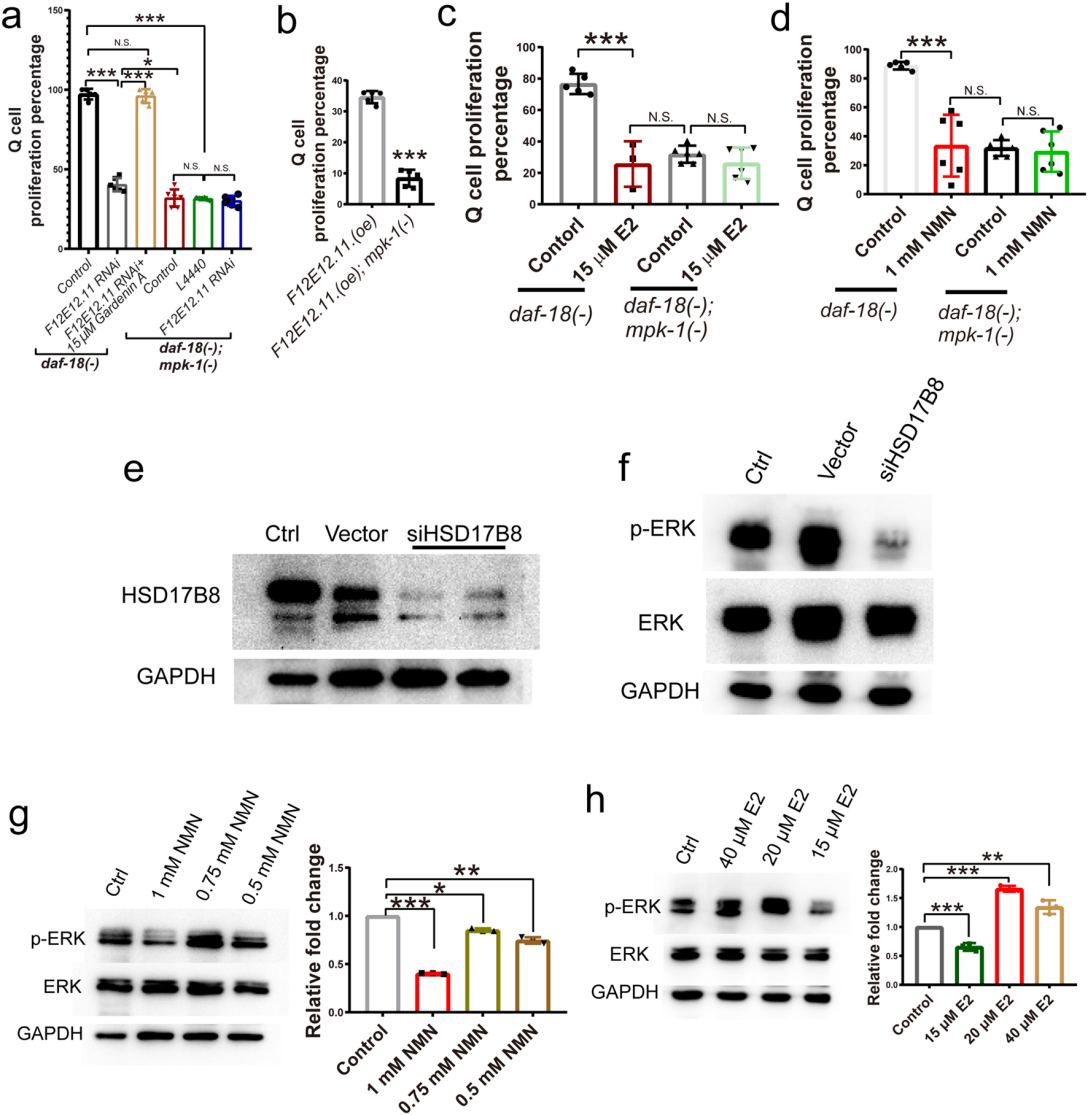
*F12E12.11*/HSD17B8 works through MAPK/ERK. (**a**) Effects of knocking down *F12E12.11* on Q cell proliferation in *daf-18* and *daf-18;mpk-1* worms. (**b**) *F12E12.11* overexpression caused Q cell proliferation to be suppressed by *mpk-1*. (**c**) Q cell proliferation in *daf-18* and *daf-18;mpk-1* worms treated with E2. (**d**) Q cell proliferation in *daf-18* and *daf-18;mpk-1* worms treated with NMN. (**a**–**d**) The test was repeated at least 3 times, with a sample size larger than 60 in each repeat, error bar: mean ± SEM. (**e**) Knockdown of the *F12E12.11* homolog gene HSD17B8 in MCF-7 cells. (**f**) Knockdown of HSD17B8 reduced the phosphorylation level of ERK. The phosphorylation of ERK was reduced by treatment with NMN (**g**) or E2 (**h**) in a dose-dependent manner. (**g**–**h**) The test was repeated 3 times, error bar: mean ± SEM. N.S.: no significant difference. *: *P* < 0.05, **: *P* < 0.01, ***: *P* < 0.001, t-test.

Previously, we found that Q cell proliferation caused by *daf-18* loss in L1-arrested worms is regulated by downstream mitogen-activated protein kinase 1 (*mpk-1*)^6^. To test whether the role of *F12E12.11* in cell proliferation depends on mpk-1, we used RNAi to knock down *F12E12.11* in *daf-18*(−) and *daf-18*(−); *mpk-1*(−) double mutants. The results showed that knocking down *F12E12.11* reduced Q cell proliferation in *daf-18*(−) mutants but failed to further suppress Q cell proliferation in *daf-18*(−); *mpk-1*(−) double mutants (Fig. 2a). The suppressive effect of *mpk-1* on Q cell proliferation in *daf-18*(−) worms was stronger than that in *F12E12.11* RNAi worms (Fig. 2a). The MPK-1/MAPK activator Gardenin A^18,19^ was used to treat the *daf-18*(−); *F12E12.11* RNAi worms; we found that activating MAPK is sufficient to compensate for the loss of *F12E12.11* (Fig. 2a). These results suggested that *F12E12.11* might work through *mpk-1* to regulate Q cell proliferation in *daf-18-*deficient worms. To further confirm the effects of *F12E12.11* on Q cell proliferation, *F12E12.11* was overexpressed in *mpk-1*(−) mutants. We found that Q cell proliferation induced by *F12E12.11* overexpression was significantly suppressed in *mpk-1*(−) mutants (Fig. 2b), indicating that the role of *F12E12.11* in Q cell proliferation in L1-arrested worms is dependent on *mpk-1*.

The *F12E12.11* gene is predicted to encode an ortholog of human NAD-dependent 17-beta-hydroxysteroid dehydrogenase, the primary function of which is converting E2–E1^9^. Reduction of E2–E1 by 17-beta-hydroxysteroid dehydrogenase was reported to increase breast cancer cell growth^12^. We treated *daf-18*(−) worms with E2, and our results showed that Q cell proliferation was significantly suppressed by 15 µM E2 treatment (Fig. 2c). The protein sequence of *F12E12.11* was blasted using BLASTP (NCBI), and the best match in *H. sapiens* was found to be estradiol 17-beta-dehydrogenase 8 (HSD17B8). HSD17B8 is an NAD-dependent dehydrogenase, and the cytosolic NAD + is converted from nicotinamide mononucleotide^20–22^. NMN was also administered to *daf-18*(−) mutants, and we found that 1 mM NMN significantly suppressed Q cell proliferation in *daf-18*-deficient L1-arrested worms (Fig. 2d).

Sex hormones, including testosterone and E2, originate from pregnenolone, a precursor to steroid hormones^23^. Enzymes in various branches of steroid hormone biosynthesis determine the conversion of pregnenolone into its derivatives. The conversion to any one of its derivatives is determined by the specific enzymes that act in different carbon arms of steroid hormone biosynthesis. Specifically, 17β-hydroxysteroid dehydrogenase/reductases play a crucial role in the biosynthesis of 19-carbon steroids, such as testosterone and E2^23,24^. Research has demonstrated that *F12E12.11* in the intestine regulates the biosynthesis of 17β-diol, impacting learning and feeding behaviors^24^. However, conversion of dehydroepiandrosterone into 17β-diol in the nervous system cannot be ruled out, as *F12E12.11* also may be expressed in neurons. Our study revealed that *F12E12.11* in the Q neuron cell lineage facilitates the conversion of E2 to E1. Notably, 17β-diol acts as a ligand for estrogen receptor-β (ER-β), a nuclear hormone receptor crucial for the actions of estrogen, particularly 17β-estradiol. Given that ER-β possesses antitumor properties, as evidenced by previous research^25^, our findings align with this by demonstrating that reducing E1 levels through knocking down *F12E12.11* suppresses the proliferation of ER-positive breast cancer cells. There are many genes encoding the large 17-beta-hydroxysteroid dehydrogenase family in *C. elegans* and humans; however, not all of them play the same role in animals. Some previous studies also showed that individual HSD17 has a specific role in enzyme activity and regulating the function of cell proliferation^12,24^.

According to our study, MPK-1/ERK works downstream of DAF-18 and HDS17B8, so loss of *mpk-1* should abolish the suppressive effects of E2 and NMN treatment on cell proliferation. Next, we treated *daf-18*(−); *mpk-1*(−) double mutants with E2 and NMN. Our results showed that E2 and NMN treatment indeed failed to further suppress Q cell proliferation (Fig. 2c–d), suggesting that the effects of E2 and NMN were abolished by loss of *mpk-1*. We predicted that the role of *F12E12.11* in cell proliferation may involve *mpk-1*, which encodes an ERK ortholog mitogen-activated protein (MAP) kinase^26,27^. Estrogen might be involved in cell proliferation in *daf-18*/PTEN-deficient animals, so ER + breast cancer cell lines^28,29^ were used in this study. The ER + breast cancer cell lines were screened, and MCF-7 cells (Michigan Cancer Foundation-7, MCF-7, human breast cancer cell line) were found to possess high levels of PTEN and HSD17B8 proteins. We knocked down the expression of HSD17B8 in MCF-7 cells using RNAi to assess the activation of ERK1/2. We found that the phosphorylated ERK level was significantly reduced by siHSD17B8 in the MCF-7 breast cancer cell line (Fig. 2e–f). To further confirm that the effect of HSD17B8 on Q cell proliferation is dependent on MPK-1/ERK, we also tested the phosphorylation levels of ERK in MCF-7 cells treated with E2 or NMN. Our results showed that activated ERK levels were significantly reduced in 1 mM NMM- or 15 μM E2-treated MCF-7 cells (Fig. 2g–h). Together, our results suggest that the roles of *F12E12.11* and its ortholog HSD17B8 in cell proliferation are dependent on MPK-1/ERK.

### E2 treatment and HSD17B8 knockdown suppress MCF-7 cell growth

**Figure 3:**
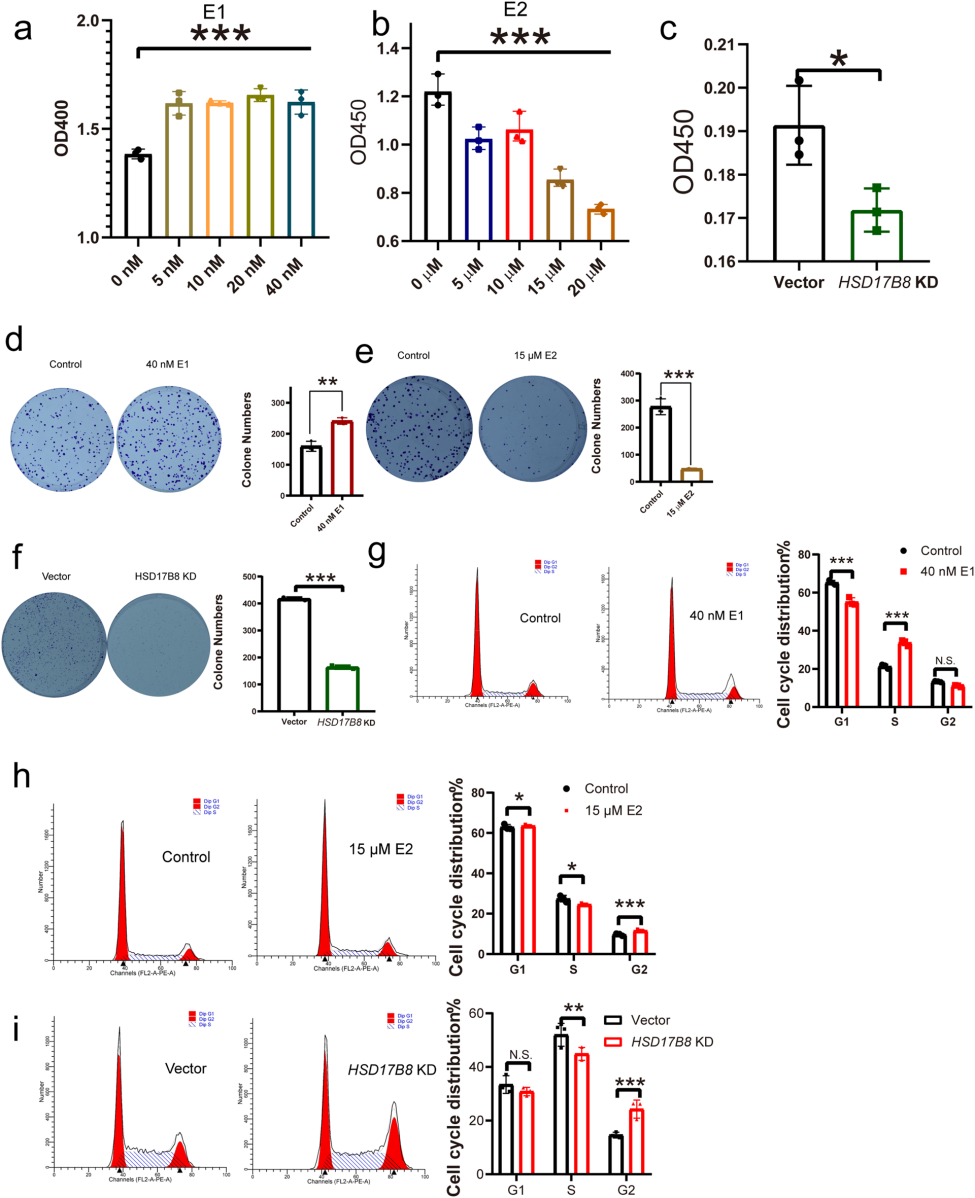
Cell proliferation and the cell cycle are affected by HSD17B8. (**a**) Treatment with E1 promoted the growth of MCF-7 cells. (**b**) Treatment with E2 suppressed the growth of MCF-7 cells. (**c**) Knocking down HSD17B8 suppressed the growth of MCF-7 cells. The colony formation assay confirmed the effect of E1 (**d**), E2 (**e**), and HSD17B8 knockdown (**f**) on MCF-7 cell growth. The cell cycle of MCF-7 cells was analyzed by using a flow cytometer after treatment with E1 (**g**) or E2 (h) or HSD17B8 knockdown (**i**). For all experiments, at least 3 biological replicates were performed. *P < 0.05; **P < 0.01; ***P < 0.001, two-sided Student’s t test. N.S.: no significant difference.

By analyzing patient tissues, we evaluated the roles of HSD17B family members in human breast cancers^12,30,31^. Another 17-beta-hydroxysteroid dehydrogenase, HSD17B14, was reported to be involved in breast cancer cell growth^12^. HSD17B family dehydrogenases oxidize E2 to E1. While E1 stimulates breast cancer cell proliferation, E2 has the opposite effect. To further test the effect of HSD17B8 on tumor cell growth, we treated MCF-7 cells with E1 and E2. The cell growth assay results showed that the growth of MCF-7 cells treated with E1 was promoted (Fig. 3a), while E2 inhibited the growth of MCF-7 cells (Fig. 3b). Our results showed that HSD17B8 knockdown significantly suppressed the growth of MCF-7 cells (Fig. 3c). We also performed a cell colony formation assay, and our results further confirmed that E1 increased the growth of MCF-7 cells (Fig. 3d) and that E2 supplementation suppressed the growth of MCF-7 cells (Fig. 3e). Knocking down HSD17B8 in MCF-7 cells could suppress the cell growth of MCF-7 cells (Fig. 3f). E2 was reported to arrest cells in G2/M phase to suppress cell growth^32^. We treated MCF-7 cells with E1 and E2 and used a flow cytometer to analyze the cell cycle. The results showed that the number of E1-treated MCF-7 cells in S phase was increased (Fig. 3g). E2 treatment (Fig. 3h) and HSD17B8 knockdown (Fig. 3i) decreased the percentage of cells in S phase and increased the percentage of cells in G2/M phase. These results suggest that HSD17B8 knockdown and the consequent accumulation of E2 can arrest tumor cells at G2/M phase. This is consistent with previous reports that PTEN promotes G2/M arrest *in vitro*^33–35^. We previously reported that loss of *daf-18*/PTEN can arrest somatic Q neuroblasts in G1/S phase of the cell cycle during L1 arrest *in vivo*^6^. PTEN has also been reported to control G1/S cell cycle arrest in breast cancer cells^36^. However, PTEN is known to regulate multiple cell cycle checkpoints, including the G1, S, G2, and M checkpoints^37–41^. These results suggest that the function of PTEN in cell cycle regulation is based on the cell type, culture conditions, and specific regulation mode. In our study, we found that PTEN physically interacts with HSD17B8 to inhibit the conversion of E2–E1 by HSD17B8; thus, the inhibitory effect of HSD17B8 blocking on cell proliferation is mainly achieved through the accumulation of E2 and a reduction in E1 levels. This may explain why knocking down HSD17B8 arrests MCF-7 cells in G2/M phase, which is also consistent with previous reports that E2 can arrest tumor cells in G2/M phase. We speculate that E2 or HSD17B8 knockdown can arrest MCF-7 cells in G2/M phase, at least in vitro.

### PTEN interacts with and impairs the function of HSD17B8

**Figure 4:**
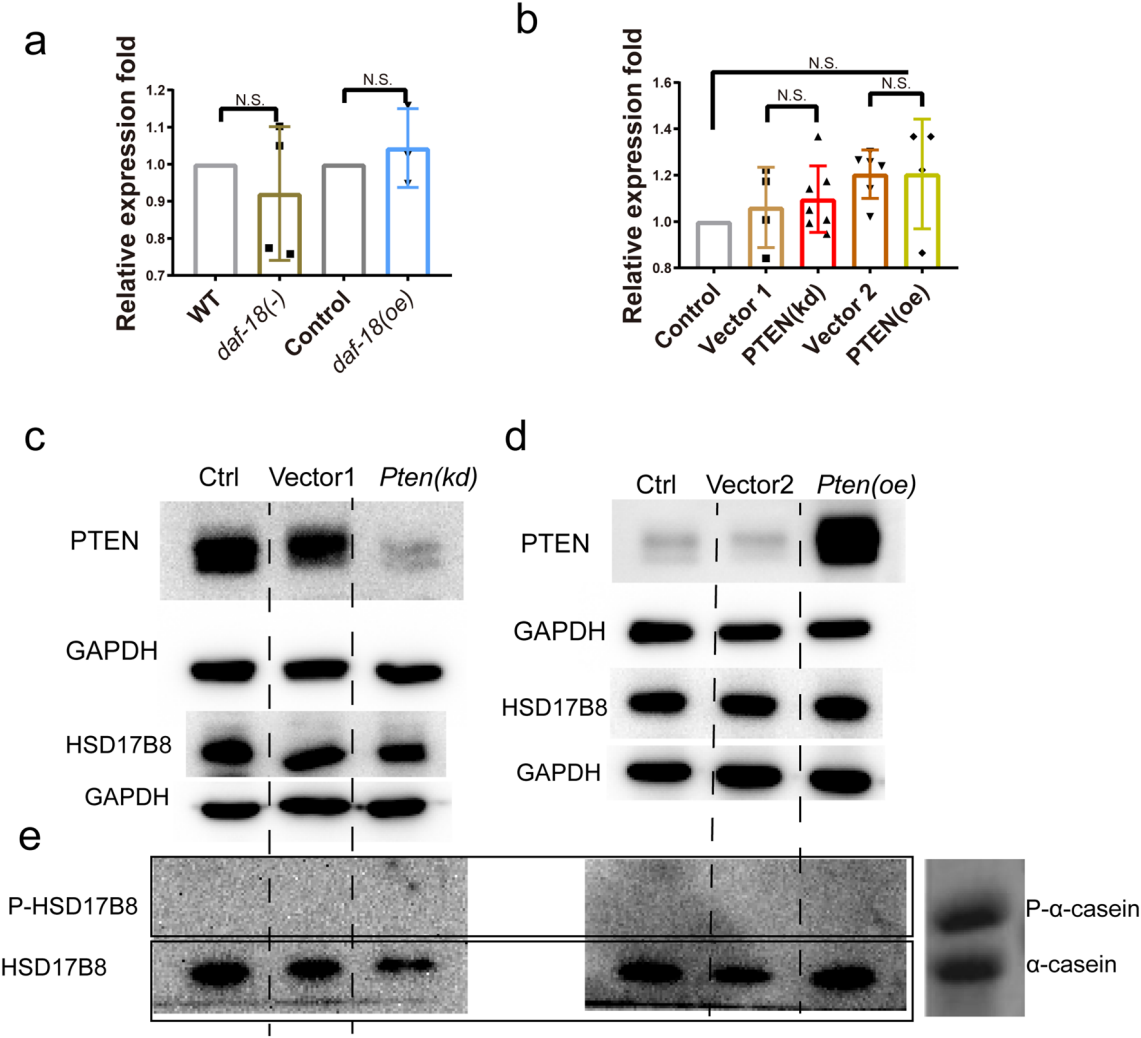
DAF-18/PTEN does not affect the translation, transcription, or phosphorylation of *F12E12.11*/HSD17B8. (**a**) Expression of *F12E12.11* in worms when *daf-18* was knocked out or overexpressed. (**b**) Expression of HSD17B8 in MCF-7 cells when PTEN was knocked down or overexpressed. (**a**–**b**) The test was repeated 3 times, error bar: mean ± SEM. (**c**–**d**) HSD17B8 protein expression in MCF-7 cells when PTEN was knocked down or overexpressed. (**e**) Phosphorylation of HSD17B8 when TEN was knocked down or overexpressed was assessed by using a Phos-tag gel. Vector 1: empty knockdown vector. Vector 2: empty overexpression vector. N.S.: no significant difference. α-Casein and phosphorylated α-casein (P-α-casein) were used as positive controls. N.S.: no significant difference. *: *P* < 0.05, **: *P* < 0.01, ***: *P* < 0.001.

**Figure 5:**
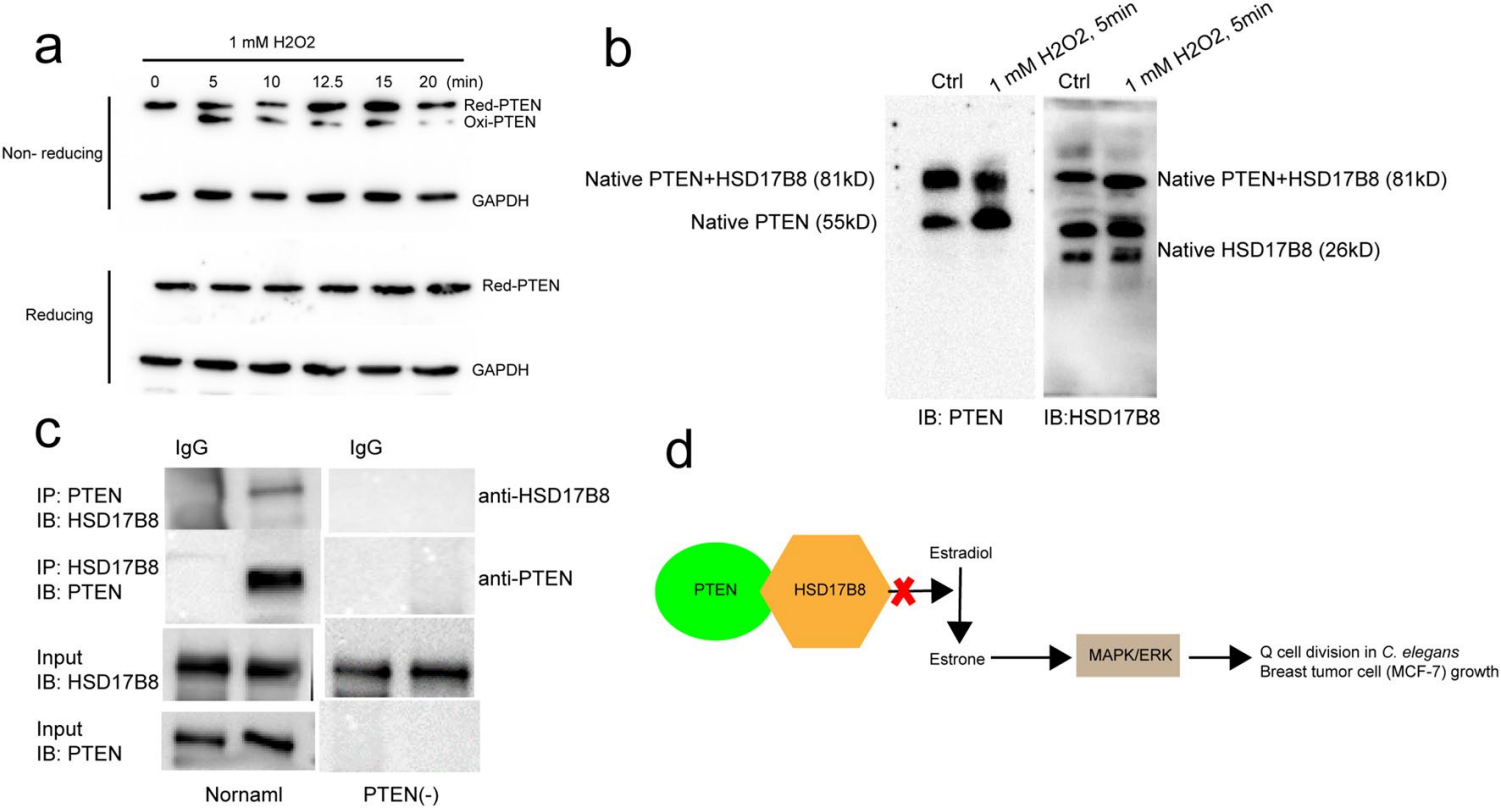
PTEN interacts with HSD17B8. (**a**) Serum-starved MCF-7 cells were treated with 1 mM H2O2. All samples were alkylated with 10 mM NEM and subjected to nonreducing or reducing SDS‒PAGE. Reduced and oxidized PTEN levels were measured. (**b**) Interaction between HSD17B8 and PTEN was tested by using native PAGE. (**c**) Co-IP was used to test the physical interaction between PTEN and HSD17B8. PTEN(−): MCF-7 cell line with the strongest PTEN knockdown achieved using RNAi. (**d**) Proposed mechanism of how PTEN regulates HSD17B8 to affect cell proliferation. PTEN does not affect HSD17B8 transcription, translation, or phosphorylation. PTEN can physically interact with HSD17B8, which may affect its function in producing estrone from estradiol. Estradiol can block cell proliferation. Loss of estradiol and accumulation of estrone can induce the phosphorylation of ERK to promote cell proliferation.

Next, we wanted to determine how *daf-18* affects the activity of *F12E12.11*. First, we tested whether the expression of *F12E12.11* is regulated by *daf-18*. The expression levels of *F12E12.11* were tested in *daf-18*(−) and *daf-18*(oe) worms, and we found that *F12E12.11* expression levels were not significantly different between these worms (Fig. 4a). We also tested the expression levels of the *F12E12.11* homolog HSD17B8 in MCF-7 breast cancer cells with knockdown or overexpression of PTEN, a *daf-18* homolog. Our results showed that the expression levels of HSD17B8 were not significantly changed in PTEN-knockdown or PTEN-overexpressing MCF-7 cells (Fig. 4b). These results suggested that *daf-18*/PTEN does not affect *F12E12.11*/HSD17B8 at the transcriptional level. Next, we also evaluated the protein level of HSD17B8. Our results showed that the protein level of HSD17B8 was not changed in either PTEN-knockdown or PTEN-overexpressing MCF-7 cells (Fig. 4c-d). PTEN is widely known as a potent tumor suppressor, and its main function is as a phosphatase^13,42–44^. As a dehydrogenase enzyme^9,45^, HSD17B8 has not been reported to be regulated by a kinase or phosphatase. PTEN was also reported to have protein phosphatase activity^42,46^. To test whether the phosphatase PTEN can regulate the phosphorylation of HSD17B8, phosphorylated HSD17B8 levels were analyzed using a published standard method (Phos-tag gels, a gel copolymerized with Phos-tag™ acrylamide for phosphorylated protein analysis)^47^. Our results showed that no form of phosphorylated HSD17B8 was detected in control, PTEN-knockdown, or PTEN-overexpressing MCF-7 cells (Fig. 4e), suggesting that PTEN does not affect the phosphorylation of HSD17B8 or that HSD17B8 is not activated through phosphorylation. All these results show that PTEN does not affect the activity of HSD17B8 in a conventional manner. We speculate that PTEN may interact with HSD17B8 to inactivate it. The PTEN active-site cysteine can be oxidized by cellular reactive oxygen species (ROS) to restrain its phosphatase activity^48–50^. An in vitro protein binding affinity analysis was performed to identify the binding partners of the purified H_2_O_2_-oxidized form PTEN, and HSD17B8 was one of the 97 potential protein interactors identified by using LC–MS (Liquid Chromatograph Mass Spectrometer) in that study^48^. This suggested that PTEN oxidation, which abolishes the phosphatase activity of PTEN, may also control the other functions of PTEN. Additionally, considering that L1-arrested worms also have higher oxidation levels than normally cultured worms^2,51^, we tested whether oxidized PTEN interacts with HSD17B8. H_2_O_2_ was used to generate oxidized PTEN, and we found that MCF-7 cells treated with 1 mM H_2_O_2_ for 5 to 15 min exhibited high levels of oxidized PTEN (Fig. 5a). Protein was extracted from H_2_O_2_-treated MCF-7 cells, and to maintain both the conformation and biological activity of the proteins, native PAGE^52^ was used to test the interaction of oxidized PTEN with HSD17B8. Interestingly, our results showed that PTEN interacted with HSD17B8 in MCF-3 cells regardless of whether it was oxidized by H_2_O_2_ (Fig. 5b), indicating that PTEN endogenously interacts with HSD17B8 to inactivate it. Furthermore, we used coimmunoprecipitation (Co-IP) to confirm that the protein PTEN physically interacts with HSD17B8 (Fig. 5c). The results suggested that PTEN acts as a tumor suppressor through its nonphosphatase activity and interacts with HSD17B8. This interaction may affect the function of HSD17B8 on preventing E2 from being converted to E1. Thus, a change in the E2/E1 ratio may play an important role in controlling cell proliferation (Fig. 5d).

## Methods

### *C. elegans* strains

*C. elegans* Strains Worm strains were acquired from the Caenorhabditis Genetics Center (CGC) and maintained according to previously described standard methods^16^. Mutants were crossed with P*egl-17*-mCherry (rdvIs1) worms to analyze Q cell division ^6,53^. The worms were cultured on NGM plates with *Escherichia coli* (OP50) at 20 °C. The strains used in this study were RDV55: *rdvIs1*, RB712: *daf-18(ok480)*, and SD420: *mpk-1(ga119)/dpy-17(e164)unc-79(e1068)*.

### EMS (ethyl methane sulfonate) mutagenesis and mutation identification

*rdvIs1*; *daf-18*(ok480) worms were used for genome-wide EMS mutagenesis for forward genetic screening^16^. Four screens were performed to identify mutants with no Q cell proliferation. In short, more than 800 synchronized L4 stage worms were incubated in a total volume of 4 mL of 50 mM EMS (Sigma) in M9 buffer for 4 h at 20 °C, and approximately 20,000 F3 generation embryos were placed onto NGM plates. The progenies of these worms were cultured in M9 buffer to generate L1-arrested worms, and mutants with no Q cell proliferation were identified. The selected mutants were crossed with the *rdvIs1*; *daf-18* (ok480) strain 3 times. The mutations of interest were identified using the recently published Sibling Subtraction method^54^.

### qRT‒PCR (real-time quantitative reverse transcription PCR)

To analyze the expression level of *F12E12.11*, worms of various stages were collected. HSD17B8 expression was tested in PTEN-knockdown and PTEN-overexpressing MCF-7 cells. Total RNA was extracted using a High Pure RNA Isolation Kit (Roche). Five micrograms of total RNA was used to synthesize cDNA using a High Capacity cDNA Reverse Transcription Kit (Applied Biosystems). qRT-PCR was performed using Power SYBR Green PCR Master Mix (Applied Biosystems) and an ABI 7500 system. The relative expression levels of the genes were determined using the 2^−△△CT^ method and normalized to *cdc-42* expression.

The following primers were used for qRT‒PCR:

*cdc-42*: forward: 5′- CTGCTGGACAGGAAGATTACG-3′,

reverse: 5′-CTCGGACATTCTCGAATGAAG-3′.

*F12E12.11:* forward: 5′-TCAACAAGGAGCAAAGGTAA-3′,

reverse: 5′-GTCACCACACTTCGCATATT-3′.

*HSD17B8*: forward: 5′-AATGGTTGTCGTGGTTCCAT-3′,

reverse: 5′-CAATCACTCCAGCCTTGGAT-3′.

*GAPDH*: forward: 5′-ACCACAGTCCATGCCATCAC-3,

reverse: 5′-TCCACCACCCTGTTGCTGTA-3′.

### RNAi (RNA interference) in *C. elegans*

Bacteria (HT115) were transformed with a double-stranded target RNA vector (L4440) carrying RNAi to inactivate the target gene. RNAi clones from the Horizon Discovery Library were grown according to the manufacturer’s protocol and then seeded onto NGM plates containing 1 mM isopropyl-β-D-thiogalactopyranoside (IPTG) for the formation of double-stranded RNA. The knockdown efficiencies of the RNAis were measured using qPCR. Eggs from RNAi-treated worms were transferred to M9 buffer at 20 °C for 2–3 days to generate L1-arrested worms. The percentage of worms with proliferating Q cells was determined under a fluorescence microscope (Nikon, SMZ18).

### Transgenic worm strains

To prepare *F12E12.11*-overexpressing strains, the gene sequence was amplified from C. elegans genomic DNA and placed under the control of its own promoter. The sequence was cloned and inserted into the L2528 plasmid (Addgene). The plasmid and an injection marker (*odr-1::rfp*) were injected into P*egl-17*::mCherry (rdvIs1) worms using standard microinjection methods^55^, and at least three stable lines were prepared from each injected strain.

The sequences of primers used for *F12E12.11* amplification are as follows:

*F12E12.11* promoter + genome sequence.

forward: 5′-AATTAAGCTTTCTCATATTAGCTATGTTCAAAAATCTGTCG-3′,

reverse: 5′-AATTGGTACCTCATGGCTGCTTCAAAATCTCCATCATATCA-3′.

The primer of *egl-17* promoter and *daf-18* can be found in our paper published previously^6^.

### Cell culture and cell line maintenance

MCF7 cells were purchased from ATCC (American Type Culture Collection) and cultured according to the manufacturer’s protocols. For estrogen treatment experiments, MCF-7 cells were estrogen-deprived by culture in phenol red-free medium supplemented with 5% charcoal-stripped FBS for 48–72 h. For estrogen treatment, medium containing 5% cFBS and either DMSO, E2, E1, or NMN was used.

### Generation of knock down and overexpression cell lines

Lentiviruses were produced as described previously^56^. Briefly, the PCDNA3.1 vector carrying PTEN overexpression constructs, the pLVX-shRNA2-Puro vector (System Biosciences) carrying PTEN RNAi, and vector DNAs were transfected into HEK293T cells, respectively, using Fugene HD (Roche). Culture media containing viruses were harvested after 48 h, filtered, and added to MCF7 cells in the presence of polybrene. Stably infected cells were selected by 2 mg/mL puromycin (Sigma) two days after infection.

The sequences of the PTEN constructs are as follows:

forward: 5′-GAATTCATGACAGCCATCATCAAAGAG-3′,

reverse: 5′-GGATCCATTCAGACTTTTGTAATTTGTG-3′.

The sequences of the PTEN RNAi are as follows:

forward: 5′-GATCCGAGAGATCGTTAGCAGAAACTCAAGAGTCTCTAGCAATCGTCTTTGTTTTTTG-3′,

reverse: 5′-AATTCAAAAAAAGAGATCGTTAGCAGAAACCTCTTGATCTCTAGCAATCGTCTTTGCG-3′.

For HSD17B8 knockdown experiments, siRNAs targeting HSD17B8 in MCF-7 cells and control vectors were transfected using the RNAiMAX transfection reagent (Thermo) according to the manufacturer’s protocol. Forty-eight hours after transfection, the cells were collected by scraping and subjected to western blotting. The sequences of the siRNAs and cDNAs are as follows:

5′-GUGGUUCCAUCAUCAACAUTT AUGUUGAUGAUGGAACCACTT-3′.

### Western blotting

Protein was extracted using a standard method. Cells were washed three times with ice-cold phosphate-buffered saline (PBS) and lysed on ice in RIPA Whole Cell Lysis Reagents & Enhancers (Sigma) supplemented with protease inhibitors and phosphatase inhibitors (Sigma). The proteins were separated by 12% SDS–PAGE (sodium dodecyl sulfate—polyacrylamide gel electrophoresis) and transferred to polyvinylidene difluoride membranes (PVDF). For native PAGE, the proteins were prepared in nonreducing nondenaturing sample buffer. The preparation and electrophoresis buffers used with the nondenaturing polyacrylamide gels did not contain SDS. The proteins were separated on ice. Phos-tag™ SDS‒PAGE (Wako) was performed according to the manufacturer’s protocol. The reducing and redox-PTEN assays were performed as previously described^49,50^. Serum-starved cells were exposed to 1 mM H_2_O_2_ for various durations. All samples were alkylated with 10 mM NEM and subjected to nonreducing or reducing SDS‒PAGE, followed by immunoblot analysis with an anti-PTEN antibody. The following antibodies were used: anti-PTEN (Santa Cruz, sc-7974,1:1000), anti-GAPDH (Proteintech, 60004-1), anti-HSD17B8 (Abcam, GR121807-4, 1:10000), anti-ERK, rabbit anti-PHOS-ERK (CST, #4695, #4370, 1:1000 and 1:1000), and secondary antibody (Elabscience, E-AB-1001, E-AB-1003).

### CO-IP (coimmunoprecipitation)

For Co-IP and western blotting, cells were lysed in 2 ml of RIPA buffer supplemented with 0.5 mM PMSF and protease inhibitors (Sigma). The cleared lysate was incubated with a specific antibody overnight at 4 °C on a rotating shaker, followed by incubation with protein A-Sepharose beads for 2 h. The samples were boiled for 5 min, and the denatured samples were electrophoresed on 7.5% Criterion SDS‒PAGE gels (Bio-Rad, Germany) and transferred to nitrocellulose membranes (Whatman GmbH, Germany). Immunoprecipitated proteins were detected with either an anti-PTEN or anti-HSD17B8 primary antibody and secondary antibodies (anti-mouse HRP 31,450, Pierce).

### Analysis of cell proliferation in *C. elegans*

In brief, embryos were maintained and hatched in sterile M9 buffer and incubated at 20 °C to initiate L1 arrest. The final Q cell descendants (A/PVM) were observed under an Axioplan fluorescence microscope (Zeiss, Germany) after 2–3 days in L1 arrest. Fifty microliters of M9 buffer containing more than 50 L1-arrested worms were collected. The total number of worms and the number of worms with proliferating A/PVM cells were counted.

### Cell proliferation assay

Cell proliferation rates were determined by the Cell Counting Kit-8 (CCK8) assay (Coolaber Biotechnology, Beijing, China) following the manufacturer’s instructions. Specifically, cells at a density of 5 × 104/mL (5000 cells/well) were seeded into 96-well plates and cultured for 14 h with complete MCF-7 medium (Procell Biotechnology, Wuhan, China). In the treatment groups, the medium was replaced with medium containing E1 or E2 (Sigma) at different concentrations, and the cells were incubated for 48 h. After the addition of 10 µL CCK-8 reagent to each well and incubation for 2 h, the absorbance at 450 nm was measured.

### Colony formation assays

After MCF-7 cells were plated in six-well plates at a density of 1000 cells/well and allowed to adhere, culture medium containing 40 nM E1 or 15 μM E2 was added for incubation for 48 h, followed by a 7 day incubation period. When cell colonies were visible under a microscope, they were washed with PBS, fixed in methanol for 20 min, and stained with 0.1% crystal violet solution for 30 min at RT. Finally, the colonies were photographed and counted.

### Cell cycle analysis by flow cytometry

Cells were seeded in a six-well plate and incubated for 24 h at 37 °C. After the cells reached 70% confluency, 40 nM E1 or 15 μM E2 was added for 48 h. A Cell Cycle Staining kit (CCS012, Multi Sciences, Hangzhou) was used for cell cycle analysis according to the manufacturer’s instructions. The cells were analyzed by a CytoFlex instrument (Beckman Coulter, USA), and Kaluza.2.1 software was used to analyze the cell cycle.

### Statistical analyses

Sample sizes were not predetermined by using any statistical method, the experiments were not randomized, and the investigators were not blinded to the conditions during the experiments and outcome assessment. All graphed data are presented as the mean ± SEM of at least 3 biological replicates. Statistical calculations were performed using GraphPad Prism 5 (GraphPad Software). The density of the western blot bands was quantified using ImageJ. The relative expression levels of genes were determined using the 2^−△△CT^ method. Differences between two groups were analyzed using two-tailed Student’s t-test, and a *P* value < 0.05 was indicative of statistical significance (*p < 0.05, **p < 0.01, ***p < 0.001, ns = not significant). Further details are provided in the figure legends.

## Data Availability

All data are available in the main text or the supplementary information.

## Supplementary Figures

Supplementary Figures.

## References

[1] Baugh LR (2013). To grow or not to grow: nutritional control of development during Caenorhabditis elegans L1 arrest. Genetics.

[2] Baugh LR, Hu PJ (2020). Starvation responses throughout the caenorhabditis elegans life cycle. Genetics.

[3] Fukuyama M, Rougvie AE, Rothman JHC (2006). Elegans DAF-18/PTEN mediates nutrient-dependent arrest of cell cycle and growth in the germline. Curr. Biol. CB.

[4] Kaplan RE (2015). dbl-1/TGF-beta and daf-12/NHR signaling mediate cell-nonautonomous effects of daf-16/FOXO on starvation-induced developmental arrest. PLoS Genetics.

[5] Chen Y, Baugh LR (2014). Ins-4 and daf-28 function redundantly to regulate C. elegans L1 arrest. Dev. Biol..

[6] Zheng S, Qu Z, Zanetti M, Lam B, Chin-Sang IC (2018). elegans PTEN and AMPK block neuroblast divisions by inhibiting a BMP-insulin-PP2A-MAPK pathway. Development.

[7] Kutscher LM, Shaham S (2014). Forward and reverse mutagenesis in C. elegans. WormBook Online Rev. C. elegans Biol..

[8] Lukacik P (2007). Structural and biochemical characterization of human orphan DHRS10 reveals a novel cytosolic enzyme with steroid dehydrogenase activity. Biochem. J>.

[9] Ohno S, Nishikawa K, Honda Y, Nakajin S (2008). Expression in E. coli and tissue distribution of the human homologue of the mouse Ke 6 gene, 17beta-hydroxysteroid dehydrogenase type 8. Molecular cell. Biochem..

[10] Key TJ (2015). Steroid hormone measurements from different types of assays in relation to body mass index and breast cancer risk in postmenopausal women: Reanalysis of eighteen prospective studies. Steroids.

[11] Howlader N (2014). US incidence of breast cancer subtypes defined by joint hormone receptor and HER2 status. J. Nat. Cancer Inst..

[12] Qureshi R (2020). The major pre- and postmenopausal estrogens play opposing roles in obesity-driven mammary inflammation and breast cancer development. Cell Metabolism.

[13] Li J (1997). PTEN, a putative protein tyrosine phosphatase gene mutated in human brain, breast, and prostate cancer. Science.

[14] Mihaylova, V. T., Borland, C. Z., Manjarrez, L., Stern, M. J. & Sun, H. The PTEN tumor suppressor homolog in Caenorhabditis elegans regulates longevity and dauer formation in an insulin receptor-like signaling pathway. *Proceedings of the National Academy of Sciences of the United States of America* **96**, 7427-7432 (1999). 10.1073/pnas.96.13.7427 PMC22102 10377431

[15] Cairns P (1997). Frequent inactivation of PTEN/MMAC1 in primary prostate cancer. Cancer Res..

[16] Brenner S (1974). The genetics of Caenorhabditis elegans. Genetics.

[17] Jorgensen EM, Mango SE (2002). The art and design of genetic screens: Caenorhabditis elegans. Nat. Rev. Genetics.

[18] Alonso-Castro AJ (2020). Evaluation of the neuropharmacological effects of Gardenin A in mice. Drug Dev. Res..

[19] Chiu S-P (2013). Neurotrophic action of 5-Hydroxylated Polymethoxyflavones: 5-Demethylnobiletin and gardenin a stimulate neuritogenesis in PC12 cells. J. Agricult. Food Chem..

[20] Davila A (2018). Nicotinamide adenine dinucleotide is transported into mammalian mitochondria. eLife.

[21] Berger F, Lau C, Dahlmann M, Ziegler M (2005). Subcellular compartmentation and differential catalytic properties of the three human nicotinamide mononucleotide adenylyltransferase isoforms. J. Biol. Chem..

[22] Luongo TS (2020). SLC25A51 is a mammalian mitochondrial NAD(+) transporter. Nature.

[23] Luu-The V (2013). Assessment of steroidogenesis and steroidogenic enzyme functions. J. Steroid Biochem. Molecular Biol..

[24] Lemieux GA, Yoo S, Lin L, Vohra M, Ashrafi K (2023). The steroid hormone ADIOL promotes learning by reducing neural kynurenic acid levels. Genes Dev..

[25] Mal R (2020). Estrogen Receptor Beta (ERβ): A Ligand Activated Tumor Suppressor. Front. Oncol..

[26] Arur S (2011). MPK-1 ERK controls membrane organization in C. elegans oogenesis via a sex-determination module. Dev. Cell.

[27] Lackner MR, Kornfeld K, Miller LM, Horvitz HR, Kim SK (1993). A MAP kinase homolog, mpk-1, is involved in ras-mediated induction of vulval cell fates in Caenorhabditis elegans. Genes Dev..

[28] Hu X (2009). Genetic alterations and oncogenic pathways associated with breast cancer subtypes. Molecular Cancer Res. MCR.

[29] Dai XF, Cheng HY, Bai ZH, Li J (2017). Breast cancer cell line classification and its relevance with breast tumor subtyping. J. Cancer.

[30] Denzel, Martin S (2014). Hexosamine pathway metabolites enhance protein quality control and prolong life. Cell.

[31] Rotinen M, Villar J, Encío I (2012). Regulation of 17β-hydroxysteroid dehydrogenases in cancer: Regulating steroid receptor at pre-receptor stage. J. Physiol. Biochem..

[32] Jun D (2008). 17α-Estradiol arrests cell cycle progression at G2/M and induces apoptotic cell death in human acute leukemia Jurkat T cells. Toxicol. Appl. Pharmacol..

[33] Zhang R (2016). PTEN enhances G2/M arrest in etoposide-treated MCF-7 cells through activation of the ATM pathway. Oncol. Rep..

[34] Kandel ES (2023). Activation of Akt/protein kinase B overcomes a G2/M cell cycle checkpoint induced by DNA damage. Molecular Cell. Biol..

[35] Martin SA, Ouchi T (2008). Cellular commitment to reentry into the cell cycle after stalled DNA is determined by site-specific phosphorylation of Chk1 and PTEN. Molecular Cancer Therapeutics.

[36] Bassi C (2021). The PTEN and ATM axis controls the G1/S cell cycle checkpoint and tumorigenesis in HER2-positive breast cancer. Cell Death Diff..

[37] Puc J (2005). Lack of PTEN sequesters CHK1 and initiates genetic instability. Cancer cell.

[38] Ramaswamy, S. *et al.* Regulation of G1progression by thePTENtumor suppressor protein is linked to inhibition of the phosphatidylinositol 3-kinase/Akt pathway. *Proceedings of the National Academy of Sciences* **96**, 2110-2115, 10.1073/pnas.96.5.2110 (1999). 10.1073/pnas.96.5.2110 PMC26745 10051603

[39] Shinde SR, Gangula NR, Kavela S, Pandey V, Maddika S (2013). TOPK and PTEN participate in CHFR mediated mitotic checkpoint. Cell. Sign..

[40] He J, Kang X, Yin Y, Chao KSC, Shen WH (2015). PTEN regulates DNA replication progression and stalled fork recovery. Nat. Commun..

[41] Brandmaier A, Hou S-Q, Shen WH (2017). Cell cycle control by PTEN. J. Mol. Biol..

[42] Wang L (2018). PTEN-L is a novel protein phosphatase for ubiquitin dephosphorylation to inhibit PINK1-Parkin-mediated mitophagy. Cell Res..

[43] Lee YR, Chen M, Pandolfi PP (2018). The functions and regulation of the PTEN tumour suppressor: New modes and prospects. Nat. Rev. Molecular Cell Biol..

[44] Ortega-Molina A, Serrano M (2013). PTEN in cancer, metabolism, and aging. Trends Endocrinol. Metabol. TEM.

[45] Hiltunen JK (2019). 17B-hydroxysteroid dehydrogenases as acyl thioester metabolizing enzymes. Molecular Cell. Endocrinol..

[46] Wozniak DJ (2017). PTEN is a protein phosphatase that targets active PTK6 and inhibits PTK6 oncogenic signaling in prostate cancer. Nat. Commun..

[47] O'Donoghue L, Smolenski A (2022). Analysis of protein phosphorylation using Phos-tag gels. J. Proteomics.

[48] Verrastro I, Tveen-Jensen K, Woscholski R, Spickett CM, Pitt AR (2016). Reversible oxidation of phosphatase and tensin homolog (PTEN) alters its interactions with signaling and regulatory proteins. Free Radical Biol. Med..

[49] Zhang Y (2020). Redox regulation of tumor suppressor PTEN in cell signaling. Redox Biol..

[50] Cao J (2009). Prdx1 inhibits tumorigenesis via regulating PTEN/AKT activity. EMBO J..

[51] Bazopoulou D (2019). Developmental ROS individualizes organismal stress resistance and lifespan. Nature.

[52] Wittig I, Schagger H (2009). Native electrophoretic techniques to identify protein protein interactions. Proteomics.

[53] Chai Y (2012). Live imaging of cellular dynamics during Caenorhabditis elegans postembryonic development. Nat. Protocols.

[54] Joseph BB, Blouin NA, Fay DS (2018). Use of a sibling subtraction method for identifying causal mutations in caenorhabditis elegans by whole-genome sequencing. G3 (Bethesda).

[55] Mello CC, Kramer JM, Stinchcomb D, Ambros V (1991). Efficient gene transfer in C.elegans: extrachromosomal maintenance and integration of transforming sequences. EMBO J..

[56] Lesch HP (2008). Generation of lentivirus vectors using recombinant baculoviruses. Gene Therapy.

