# Supplementary material for: Inhibiting HSD17B8 suppresses the cell proliferation caused by PTEN failure
Source: Sci Rep. 2024 May 29;14:12280. doi: 10.1038/s41598-024-63052-5 (PMC11137105; doi:10.1038/s41598-024-63052-5)
Supplement: Supplementary file 1 — Supplementary Figures. [file 41598_2024_63052_MOESM1_ESM.pdf]

WB raw figure in the manuscript:

Figure 2e:

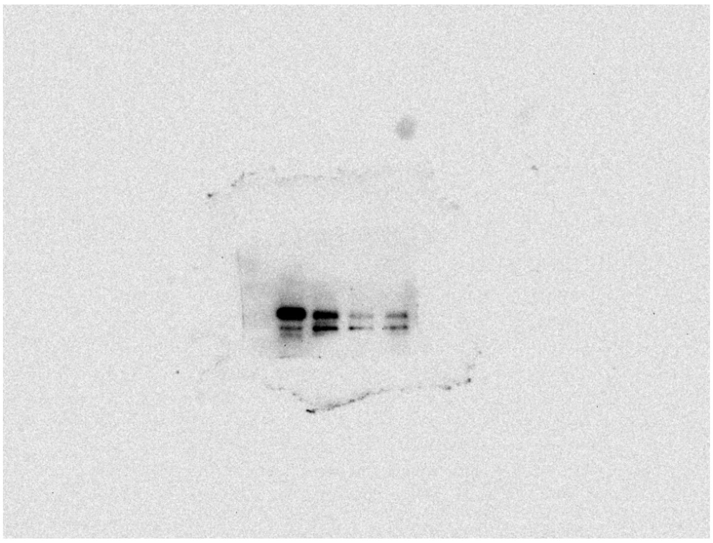

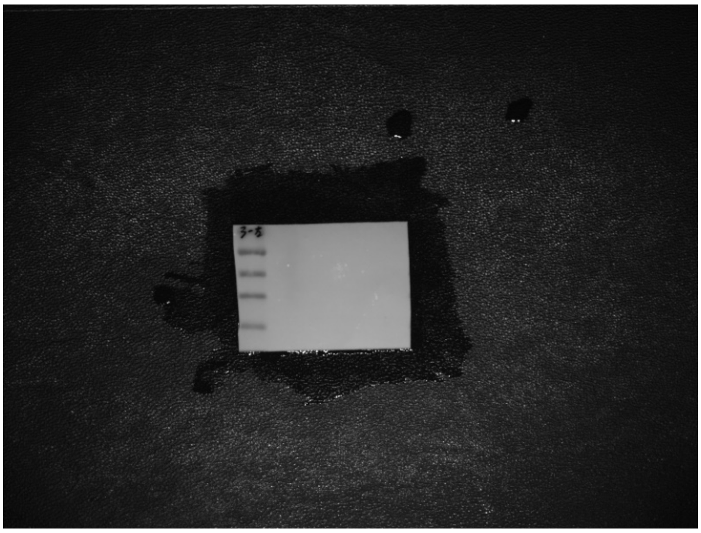

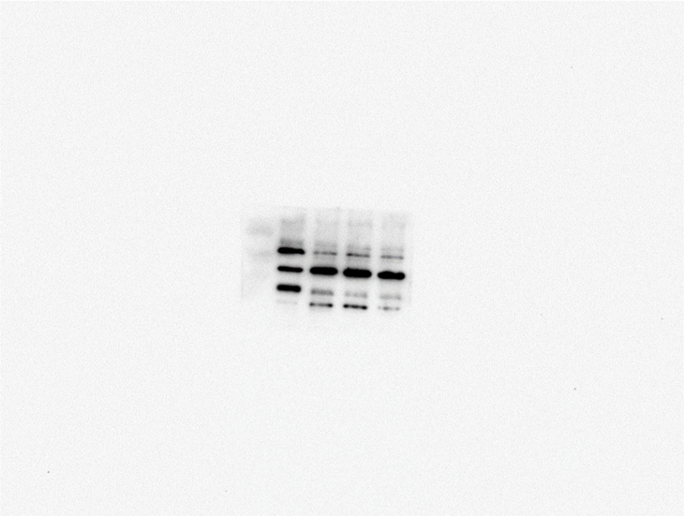

Row1: HSD178B

Row2: Marker

Row3: GAPDH

Figure 2f:

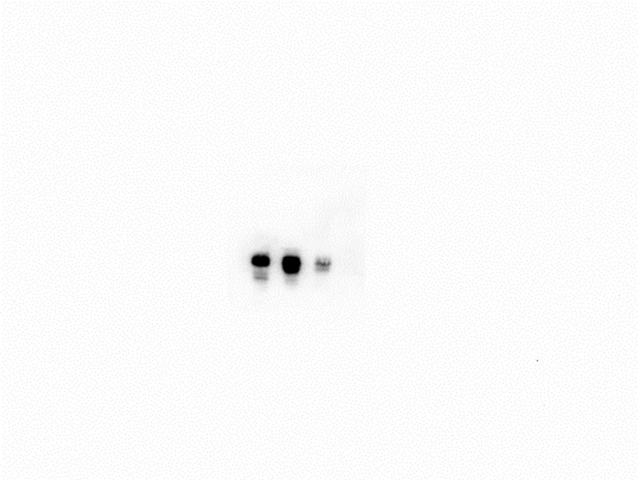

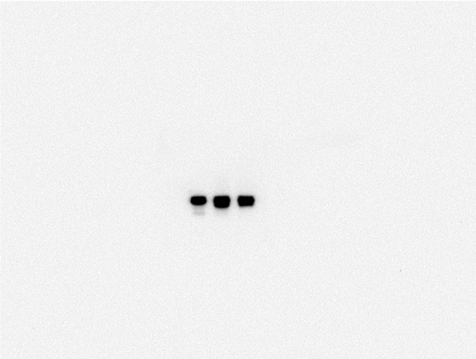

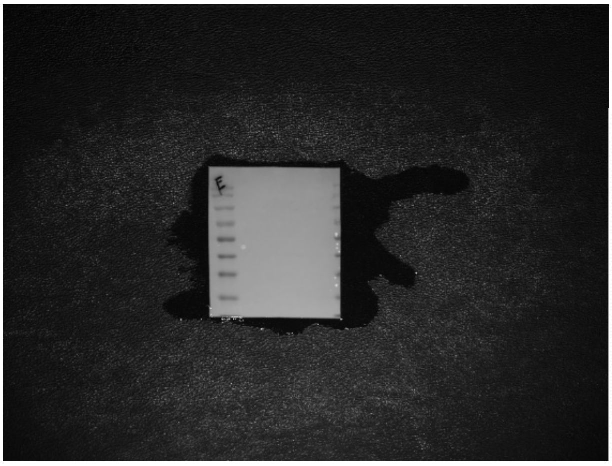

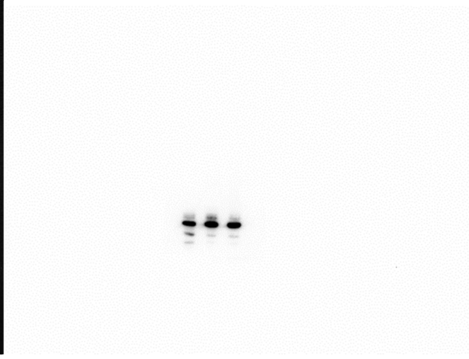

Row1: P-ERK

Row2: ERK

Row3: Marker and GAPDH

Figure 2g:

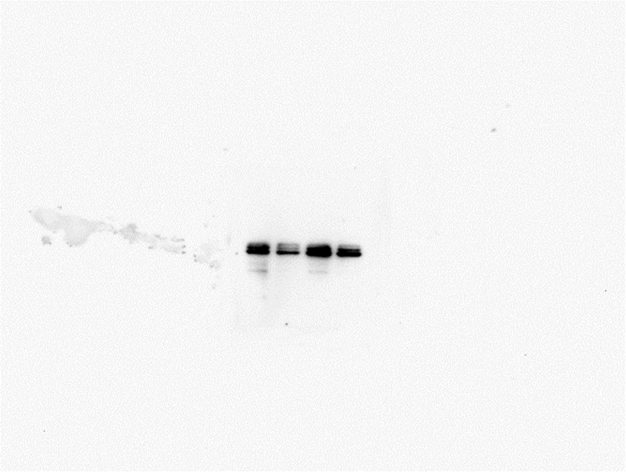

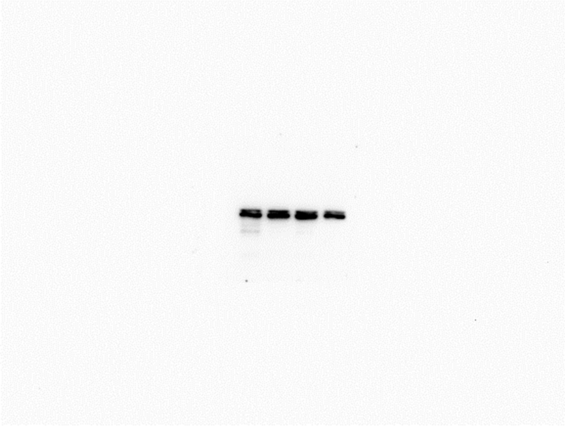

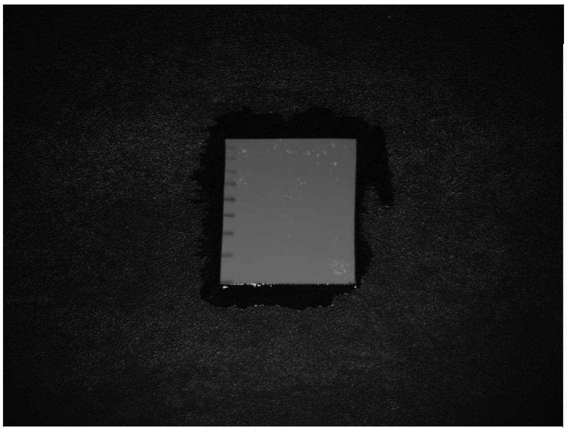
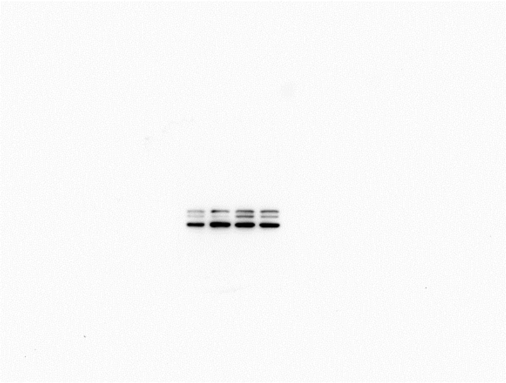

Row1: P-ERK and ERK

Row2: ERK

Row3: Marker and GAPDH

Figure 2h:

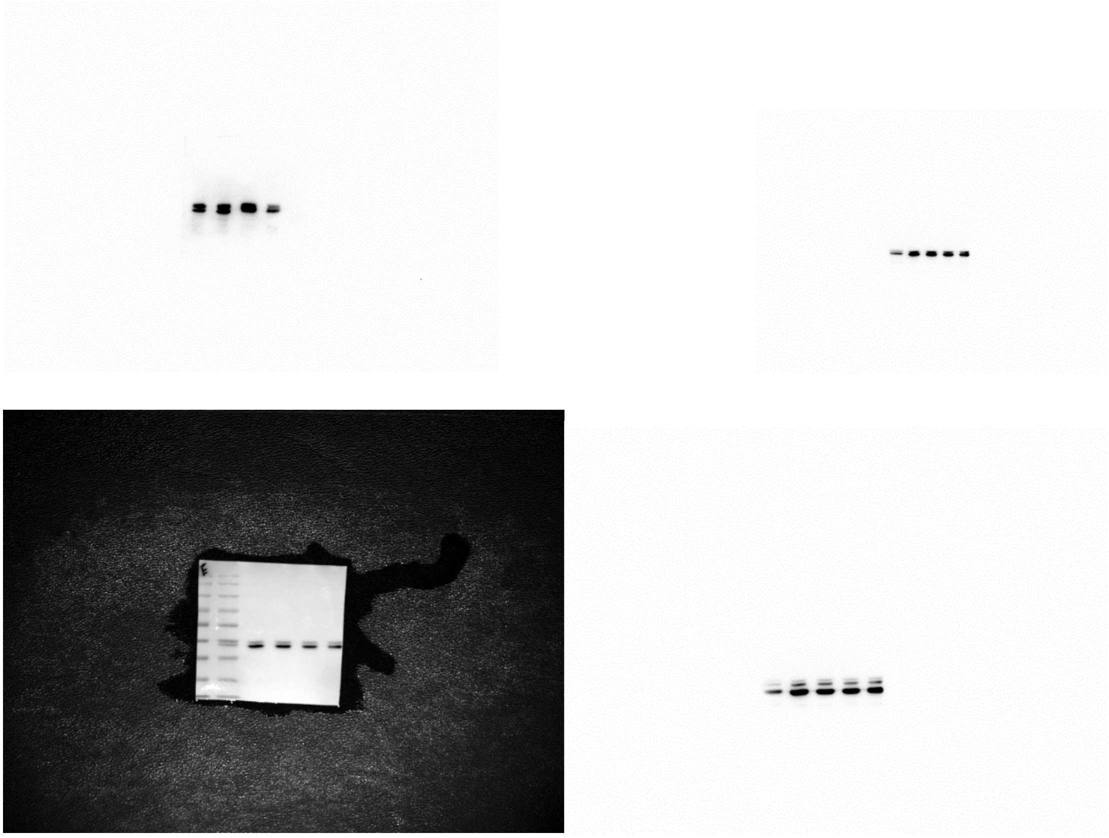

Row1: P-ERK

Row2: ERK

Row3: Marker and GAPDH

Figure 4c:

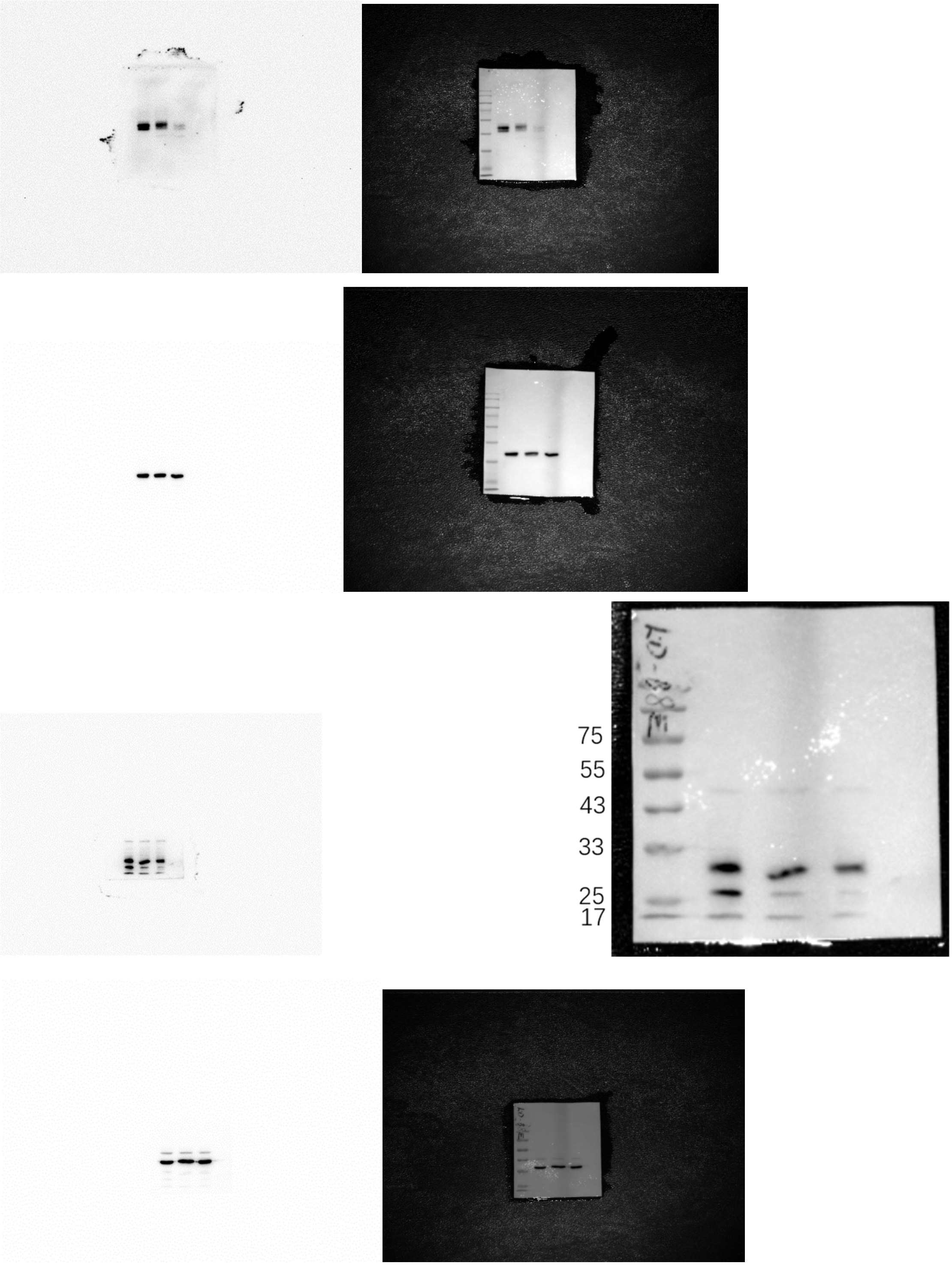

Row1: PTEN

Row2: GAPDH

Row3:HSD17B8

Row4: GAPDH

Figure 4d:

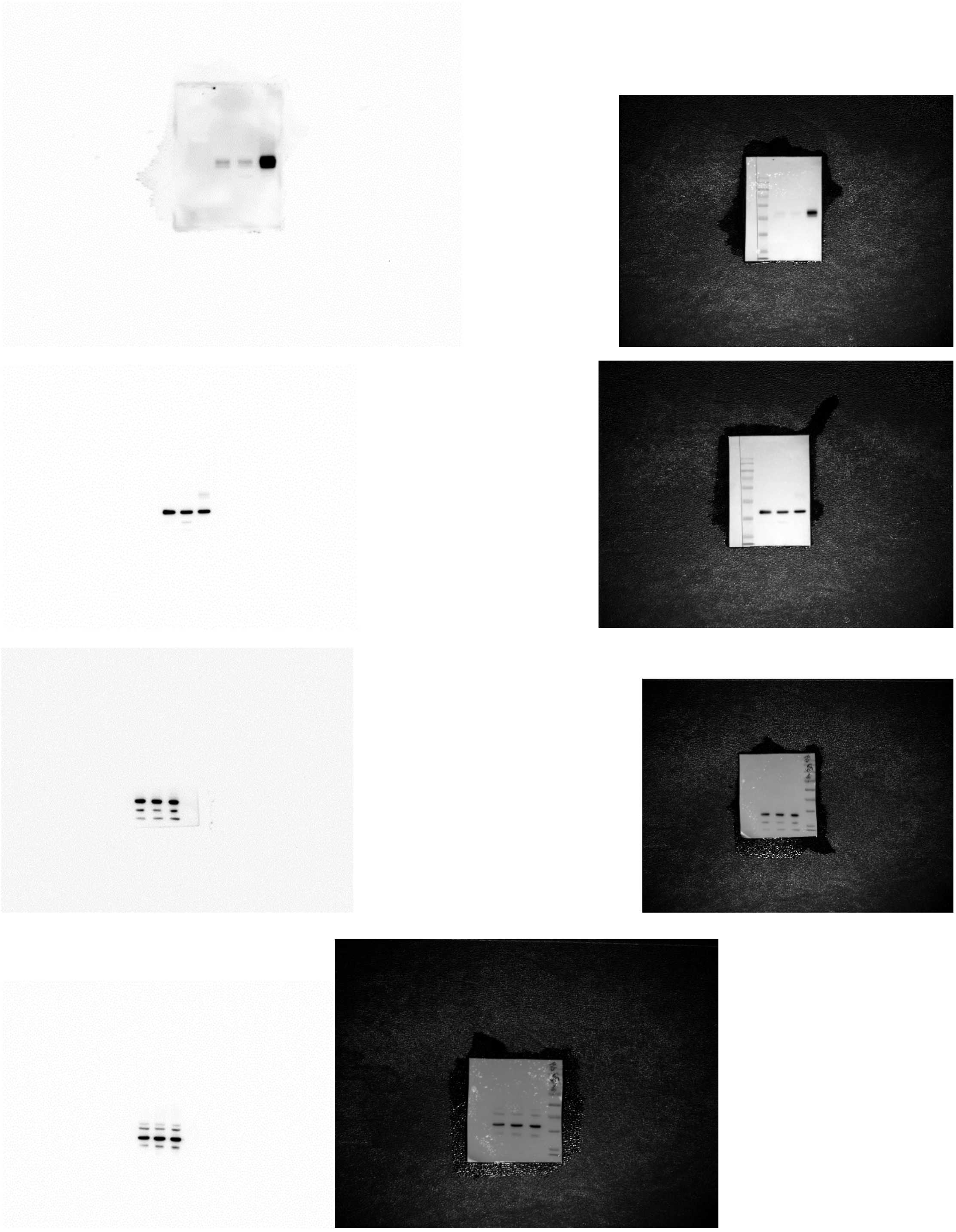

Row1: PTEN

Row2: GAPDH

Row3:HSD17B8

Row4: GAPDH

Figure 4e:

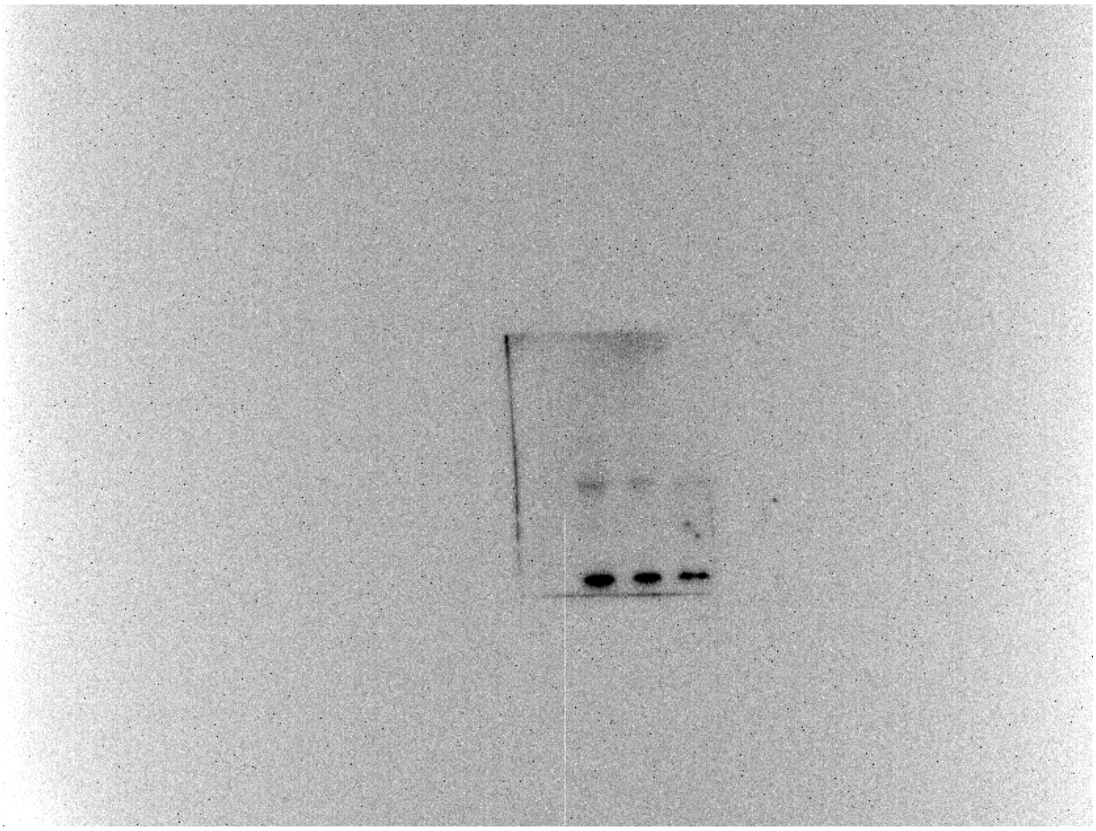

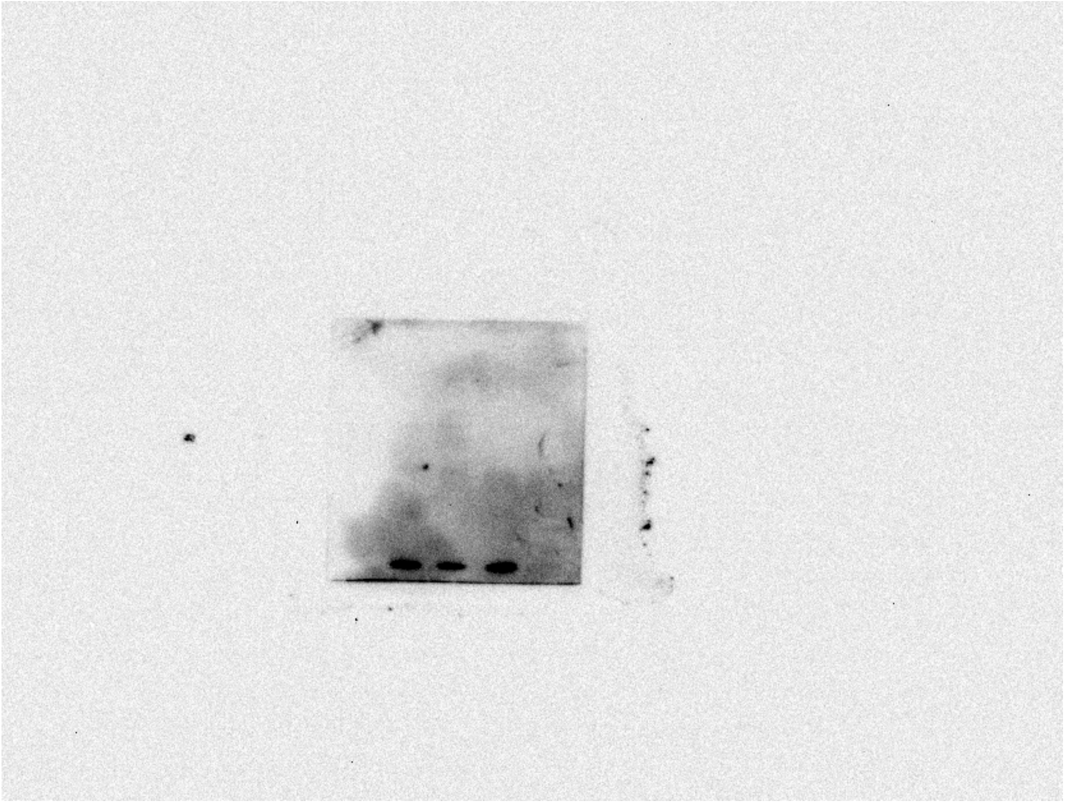

Row1: p-HSD17B8 and HSD17B8

Row2: p-HSD17B8 and HSD17B8

Figure 5a:

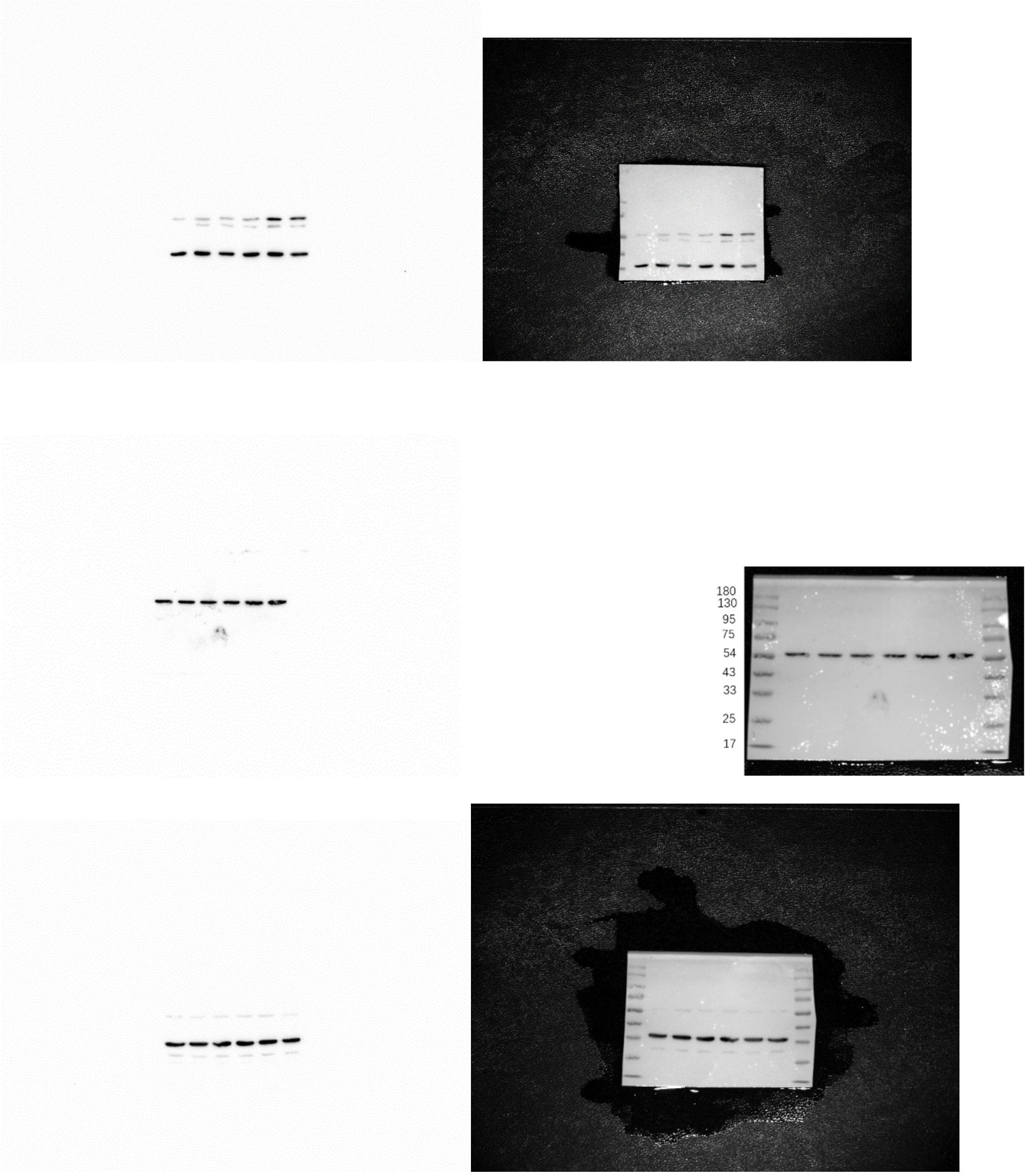

Row1: Red-PTEN and Oxi-PTEN

Row2: GAPDH

Row3:Red-PTEN and GAPDH

Figure 5b:

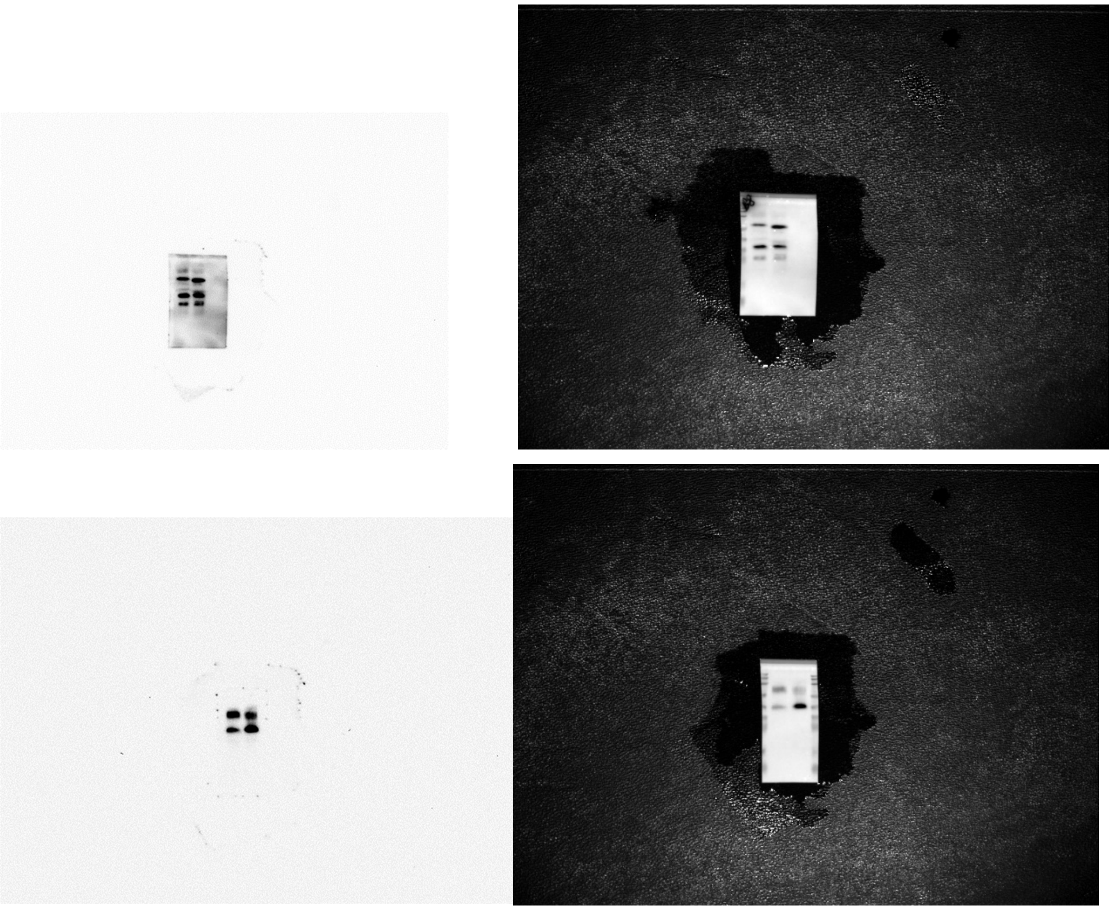

Row1: IB-HSD17B8

Row2: IB-PTEN

Figure 5c:

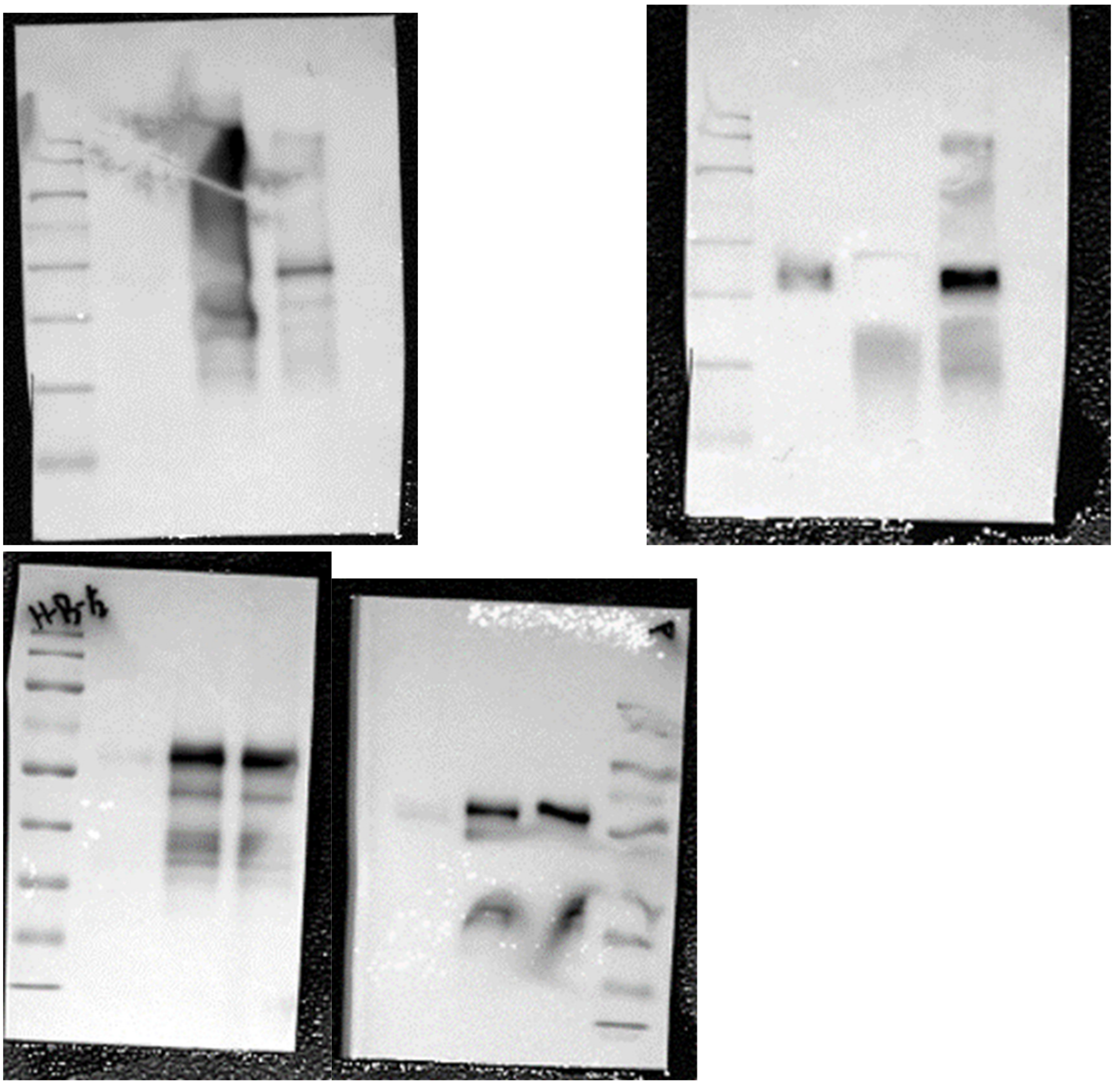

Row1: IP-PTEN, IB-HSD17B8 and IP- HSD17B8, IB-PTEN

Row2: input, IB-HSD17B8 and IB-PTEN
